# Anti-tumor Activity of Bufalin by Inhibiting c-MET Mediated MEK/ERK and PI3K/AKT Signaling Pathways in Gallbladder Cancer

**DOI:** 10.7150/jca.38393

**Published:** 2020-03-04

**Authors:** Liqiang Qian, Haoyuan Su, Gang Wang, Bin Li, Genhai Shen, Quangen Gao

**Affiliations:** Department of General Surgery, Suzhou Ninth People's Hospital, Suzhou, China

**Keywords:** Bufalin, gallbladder carcinoma, c-MET, MEK/ERK, PI3K/AKT

## Abstract

Gallbladder cancer is one of the most common malignant tumors in the biliary tract. In recent years, the chemotherapy treatment for gallbladder carcinoma has exhibited obvious characteristics of drug resistance and insensitivity, and one of the main factors is the existence of cancer stem cells. Here in this study, the effect of Bufalin on gallbladder cancer (GBC-SD) cells and the related mechanism were studied. The results indicated that Bufalin could inhibit the growth of gallbladder carcinoma both *in vivo* and *in vitro*. According to the biological behavior analysis, Bufalin induced apoptosis, inhibited the propagation, migration and invasion of GBC-SD cells, and blocked cell cycle at the G2/M stage. Besides, Bufalin inhibited the tumor sphere formation capability of gallbladder carcinoma in matrigel, reduced the expression of multiple stemness-associated proteins, including Oct4, Sox2 and the stem cell-surface marker proteins CD133 and CD44. Western blot assay showed that Bufalin inhibited MEK/ERK and PI3-K/AKT signaling pathways by inhibiting the expression of p-c-Met, which in turn affected the expression of apoptosis-related protein Mcl-1, and the invasion-associated proteins E-cadherin, MMP9 and Snail. Bufalin was found to have an inhibitory effect on the GBC-SD cell growth and reduce the self-renewal and characteristic of gallbladder cancer stem cells. It enhanced the chemotherapeutic sensitivity and reduced the metastasis of gallbladder carcinoma. In conclusion, Bufalin can be used as a new promising anticancer drug for gallbladder cancer patients who are resistant to traditional chemotherapy.

## Introduction

Gallbladder carcinoma (GBC) is the most common hepatobiliary carcinoma which is usually asymptomatic in the early stage and patients with advanced gallbladder carcinoma have no chance of radical operation. Chemotherapy is the major treatment for gallbladder carcinoma but with poor prognosis. Its early metastasis leads to a high recurrence and fatality rate, with 5-year survival rate below 10% in advanced gallbladder carcinoma patients 1. Recently, numerous papers have indicated that the key factor in the formation and development of tumors is the continuous self-renewal of cancer stem cells, which produces a resistance to chemotherapy and ultimately leads to the tolerability of chemotherapy. Shi et al. cultured CD133^+^ cell spheres from human primary gallbladder cancer cells and the GBC-SD cell line using the serum-free suspension culture technique 2. The cell spheres have a strong proliferative capacity *in vitro* and can form new subcutaneous xenograft in nude mice, showing the self-renewal property of cancer stem cells. Therefore, the search for chemotherapy drugs for gallbladder cancer stem cells is expected to lead to a fundamental remission of gallbladder carcinoma.

Bufalin is a cardiotonic steroid (CTS) isolated from the Chinese traditional medicine toad venom, which is a toxic compound with the highest activity. Studies have shown that Bufalin has many pharmacological activities including anti-inflammatory 3, anti-tumor 4 and antiviral activities 5. Recent experimental studies have also suggested that Bufalin had an inhibitory effect on cancer stem cells (CSCs). Bufalin significantly inhibited the sphere formation capability of gemcitabine-resistant pancreatic cancer cell line, decreased the expression of CD24 and ESA, and reduced the metastatic ability of pancreatic cancer-bearing nude mice by inhibiting the Hedgehog signaling pathway, thereby inhibited the development of pancreatic cancer stem cells 6. Bufalin was also reported to regulate the expression of DNMT1 and P27 by targeting the miR-148, which inhibits the proliferation and differentiation of osteosarcoma stem cells 7. Chang et al. collected and cultured the CD133^+^ and CD44^+^ osteosarcoma hMG63 cells in low attachment condition. After treating with Bufalin, the sphere formation rate of cultured hMG63 cells was decreased and the expression of Oct4 and CD133 within sphere-forming cells was down- regulated 8. The above results suggest that Bufalin has a certain inhibitory effect on cancer stem cells and can significantly reduce the metastasis and recurrence rate of cancer. In addition to their stem cell properties, CSCs have become the focus of pharmacological research due to their resistance to various cancer therapies. More research is urgently needed to find effective drugs to inhibit cancer stem cells. Through the combination of drugs that inhibit cancer cells and cancer stem cells, better therapeutic effect can be achieved. Elimination of CSC may be a key factor in long-term survival of cancer patients 9. However, the effect of Bufalin on gallbladder carcinoma has not been reported. Therefore, this project studies the effect of Bufalin on the development and stemness of gallbladder cancer, and the mechanism underlying. The conclusions will provide new clues for the effective treatment of gallbladder cancer.

## Materials and methods

### Drugs and antibodies

**Figure 1:**
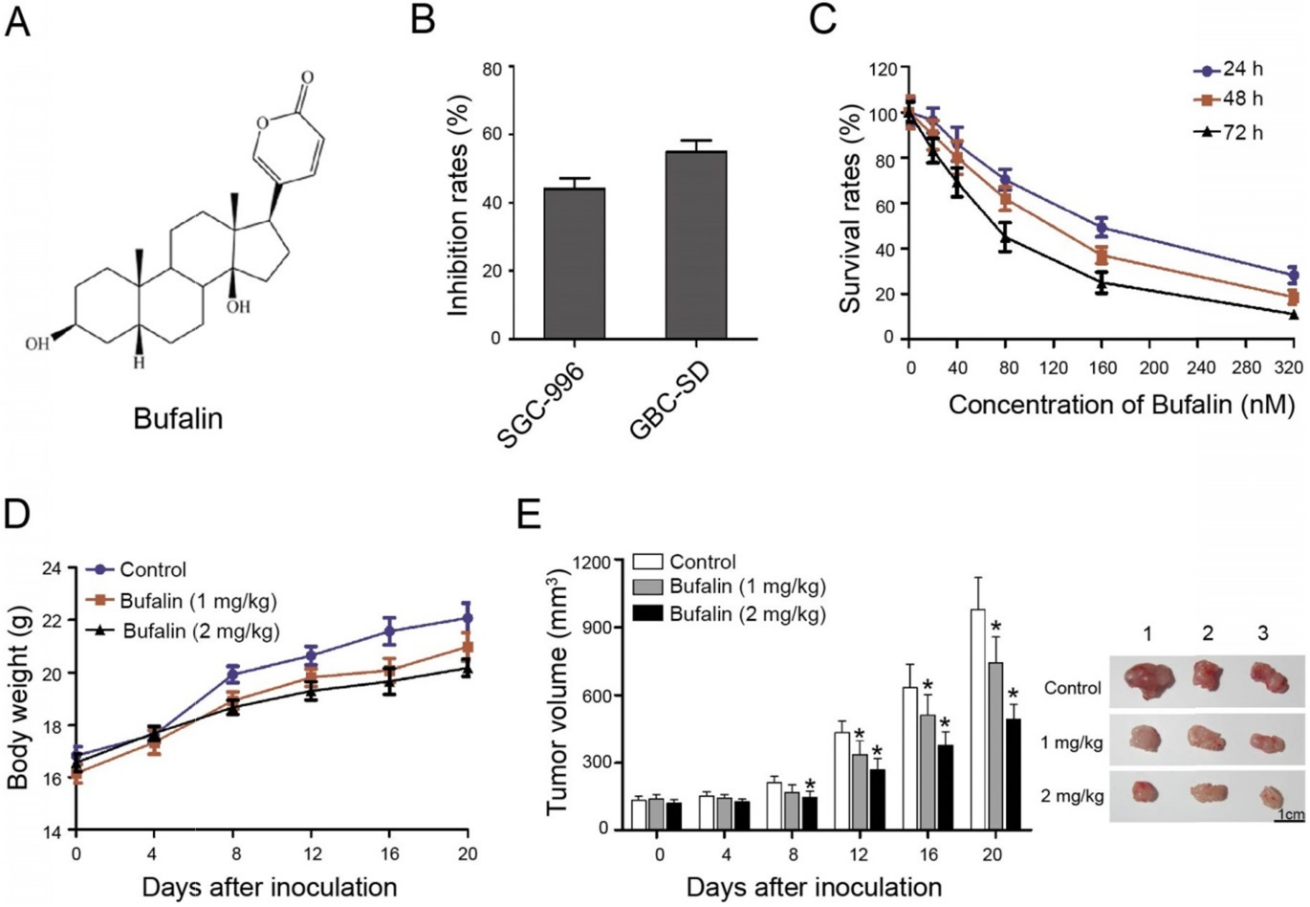
Bufalin suppressed xenografted gallbladder cancer growth *in vivo*. (**A**) Chemical structural formula of Bufalin. (**B**) The GBC-SD and SGC-996 cells were treated with Bufalin at a concentration of 100 nM for 48 hours and the inhibition rates were examined and compared; (**C**) Cell proliferation of GBC-SD cells after treatment by Bufalin at different concentrations (for 24 hours, 48 hours and 72 hours, respectively) was detected with a CCK-8 assay. (**D**, **E**) Xenograft model was obtained by subcutaneously injecting the nude mice with GBC-SD cells. When the volume of tumor is about 3 mm^3^, Bufalin at different concentrations were injected into mice by intravenous injection for 20 days. The weight and size of mice were monitored regularly during treatment. *P<0.05 *vs* Control. Bar=1 cm.

Bufalin and 5-Fu (Fig. 1A) were purchased from Yuanye biotechnology co. LTD (Shanghai). Primary antibodies include anti-Bcl-2, anti-Mcl-1, anti-Bax, anti-P21, anti-P27, anti-MMP9 and anti-MMP2 antibodies were purchased from Abcam; anti-E-cadherin, anti-Snail, anti-CD44, anti-CD133, anti-Sox2, anti-Oct4 and anti-Nanog antibodies were purchased from ABclonal; anti-AKT, anti-p-AKT, anti-p-ERK, anti- MEK, anti-p-MEK, anti-p-P38, anti-P38, anti-p-JNK and anti-JNK antibodies were purchased from CST.

### Cell lines and culture

Gallbladder carcinoma SGC-996 and GBC-SD cells were bought from the Cell Bank of Shanghai Institutes for Biological Sciences. Cells were cultured at 37°C in DMEM medium with 10% fetal calf serum (Gibco, USA) in a humidified atmosphere containing 5% CO_2_.

### Cell counting kit-8 (CCK-8) assay

The effects of Bufalin on cell proliferation of the GBC-SD cells were evaluated by Cell Counting Kit-8 (CCK-8, Beyotime Biotechnology, Shanghai, China). GBC-SD cells were cultured in 96-well plates at a concentration of 5×10^3^/100 μL per well and incubated overnight. Cells were divided into treat groups and the control group. For the treat groups, the media were substituted with fresh media containing various concentrations of Bufalin (0, 20, 40, 80, 160, 320 nM). For the control group, the 0.1% of dimethylsulfoxide (DMSO) was added instead of Bufalin. All cells were cultured for 48 h, the 10μL of CCK8 was supplied and cultured for 4 h away from light. The OD_450_ was recorded by the spectrophotometer. The cell survival rate (SR) is calculated using the formula:

SR (%) = (OD_treated group_/OD_control_) × 100%。

### Cell cycle assay

We use the flow cytometric assay to test the effect of Bufalin on GBC-SD cell cycle. Cells were seeded into 6-well plates (1.5 × 10^5^ cells/well) and cultured overnight. Bufalin at different concentrations (0, 50, 100 nM) were added and incubated for another 48 h. Then cells were digested with EDTA-free trypsin and washed twice with PBS (1,000 rpm for 3 min). Cells were fixed with precooled 70% ethyl alcohol overnight at 4°C and stained with PI (MultiSciences Biotech, Shanghai, China) for 30 min in dark before the flow cytometric assay.

### Apoptosis assay

The apoptosis of GBC-SD cells was detected by the Hoechst 33342 (Beyotime Biotechnology, Shanghai, China) staining after the Bufalin treatment. GBC-SD cells were seeded into 6-well plates (1.5 × 10^5^ cells/well) and incubated overnight. Then Bufalin at different concentrations (0, 50, 100 nM) were added and incubated for another 48 h, then cells were fixed for 20 min at RT with 4% formaldehyde. Cells were incubated with Hoechst 33342 (10 μg/mL) in the dark for 15 min and cells were observed under a fluorescence microscope.

### Migration and invasion assays

Transwell chambers (8.0 μm pore size, Millipore, Billerica, MA, USA) were utilized to determine the effects of Bufalin on GBC-SD cell invasion and migration. GBC-SD cells were digested, counted and diluted with the serum-free DMEM medium to a concentration of 3 × 10^4^ cells/200 μL. The 500 μL of medium containing 20% FBS was added into the lower chamber, while 200 μL of diluted cell suspension was added in each upper chamber. Bufalin was added into each chamber to reach a concentration of 0, 25 and 50 nM, respectively. After incubation for 48 h, cells were stained in crystal violet for 12 min, washed three times with PBS and photographed by the microscope (200 ×).

For the invasion assay, transwell chambers with matrigel were used and the concentration of 5 × 10^4^ cells/200 μL was added in each upper chamber. The rest of the steps are as described above.

### Western blot analysis

GBC-SD cells were exposed to Bufalin (0, 50, 100 μM, respectively) for 48 h and washed twice with PBS. Cells were incubated in lysis buffer on ice for 30 min and the supernatant was collected after centrifugation (12000 rpm for 30min at 4℃). The loading buffer was added and heat the mixture at 95℃ for 10 min. Samples can be stored at -80℃. Western blot was performed according to the general protocol and the ECL imaging kit (Thermo Scientific, Shanghai, China) was used to get the chemiluminescent signals. GAPDH was used as the control and the grey value was analyzed by the Image-Pro Plus 6.0.

### Low attachment sphere-forming assay

Low attachment sphere-forming assay was used to determine the effect of Bufalin on the sphere-forming capability of GBC-SD cells. GBC-SD cells treated with Bufalin (0, 50, 100 nM, respectively) were seeded at a concentration of 5 × 10^2^/100 μL in each well of the low attachment 96-well plates and cultured in DMEM supplemented with 10% of FBS, 20 ng/ml bFGF and 20 ng/ml bEGF. The cell sphere- formation was observed after culturing for 7 days and sphere-forming capability of GBC-SD cells was analyzed by micrography.

### *In vivo* efficacy of Bufalin

Procedures for animal experiments were approved by the Ethics Committee of Suzhou Ninth People's Hospital (Suzhou, China). A total of 9 male BALB/c nude mice (four-weeks old) were collected and maintained under specific pathogen-free (SPF) conditions. For each mouse the 150 μL (3×10^6^) of GBC-SD cells in were subcutaneously injected into the nude mice. The tumor-bearing mice were divided into 3 groups and treated with Bufalin (0 mg/kg, 1 mg/kg or 2 mg/kg, three mice for each concentration) by intravenous injection for 20 days. The size of tumor, weight of mice, tumor growth and mice survival were continuously observed and recorded. All mice were sacrificed after treatment and the tumors were weighed, measured and photographed. The therapeutic effect of Bufalin on gallbladder carcinoma xenograft were statistically analyzed.

### Statistical analysis

All the assays were done in triplicate, data were analyzed using the GraphPad prism version 5.0 (GraphPad Software, San Diego, CA, USA) software packages and SPSS 19.0 (SPSS Inc., Chicago, IL, USA). The grey value was analyzed by the Image-Pro Plus 6.0. The ANOVA was applied and *P* < 0.05 was considered statistically significant.

## Results

### Bufalin suppressed xenografted gallbladder cancer growth in mice

At present, there is no immortalized normal human gallbladder epithelial cell line. Normal human gallbladder epithelial cells were not necessarily used as control cells in most articles 10-14. The primary cells of human gallbladder epithelium were isolated and used as the research objects in a few articles according to their research needs 15. Since the purpose of this study is to evaluate the effect of Bufalin on gallbladder cancer cells, the results of this study mainly reflect the changes of biological characteristics of human gallbladder cancer epithelial cells before and after treatment. The effects of Bufalin on normal cells or organisms are verified by *in vivo* experiments in animals. Therefore, in this study, it is not necessary to isolate normal primary human gallbladder epithelial cells as a control group.

To detect the effect of Bufalin on gallbladder cancer cells, SGC-996 and GBC-SD cells were selected as the study object. Cells were treated with 100 nM Bufalin for 48 h. The results indicated that GBS-SD cells were more sensitive to Bufalin than the SGC-996 cells (Fig. 1B). Besides, GBC-SD cells were exposed to different concentrations of Bufalin and the results showed that the cell survival rate decreased with increasing concentration and time, indicating that Bufalin treatment was concentration- and time- dependent (Fig. 1C).

In addition, the nude mice with subcutaneous xenograft model of GBC-SD cells were prepared to study the treating effect of Bufalin on gallbladder carcinoma. The body weights of nude mice which were intravenously injected with Bufalin showed no significant difference compared with the control. The body weight of control group was slightly higher than that of the experimental group, which might be due to the tumor volumes of the control group are slightly larger, indicating that the treatment was of low toxicity (Fig. 1D). After 20 days of treatment, the volume of xenograft tumor changed obviously. Compared with the control group, Bufalin (2 mg/kg) could significantly reduce the volume of xenograft tumor by about 40 percent (Fig. 1E). These results indicated that Bufalin inhibits gallbladder cancer both *in vivo* and *in vitro*.

Because Bufalin has not been approved for clinical trials in the treatment of human gallbladder cancer, this study did not include the evaluation of any subject or any resected human tumor sample. *In vivo* experiments in nude mice with subcutaneous tumorigenesis were carried out to verify the response and toxicity of Bufalin in animals. The results can reflect the status of Bufalin *in vivo* to some extent. It is possible to apply for clinical trials only after rigorous and sufficient laboratory validation.

### Bufalin suppressed cell proliferation by inducing cell apoptosis and cell cycle arrest

**Figure 2:**
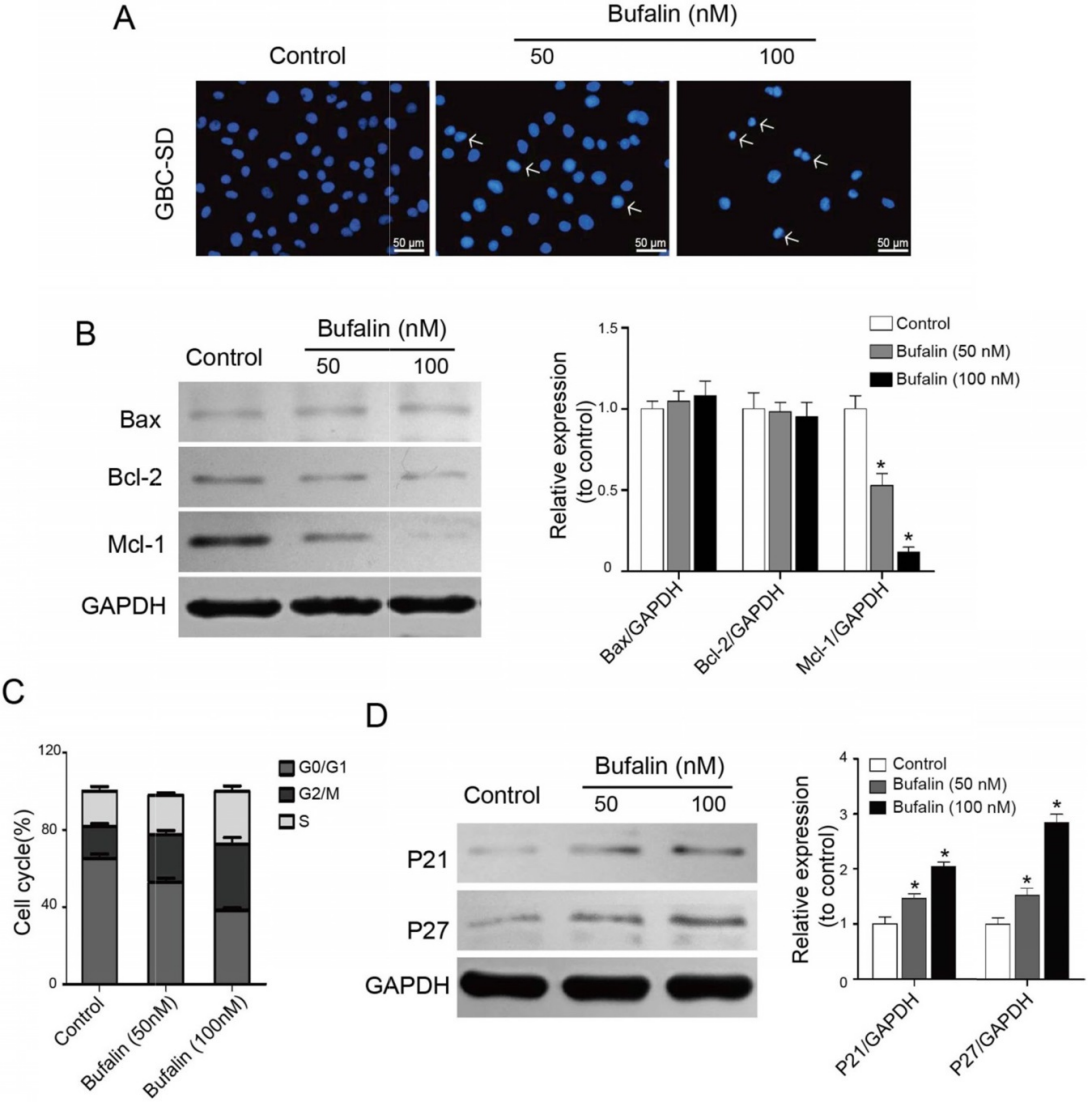
Bufalin influenced GBC-SD cell apoptosis and cell cycle distribution. (**A**) Detection of GBC-SD cell apoptosis. GBC-SD cells were treated by Bufalin for 48 hours under different concentrations (0, 50, 100 nM, respectively) and then stained with Hoechst 33342 (Bar= 50 μm). (**B**) The expression of Bcl-2, Bax and Mcl-1in GBC-SD cells were detected after 48 hours of treatment with Bufalin at different concentrations (0, 50, 100 nM, respectively) by western blot. GAPDH was the loading control (*P<0.05 vs control). (**C**) Cell cycle distribution of GBC-SD cells after treating with Bufalin was detected by flow cytometry. (**D**) The expression of P21 and P27 in GBC-SD cells were detected after 48 hours of treatment with Bufalin at different concentrations (0, 50, 100 nM, respectively) by western blot. GAPDH was the loading control (*P<0.05 *vs* Control).

To uncover the inhibitory mechanism of Bufalin on gallbladder cancer, we preliminarily studied whether Bufalin affected the apoptosis of GBC-SD cells by Hoechst staining. After 48 h of treatment, we found that the GBC-SD nucleus decreased significantly with the increase of Hoechst 33342 concentration, and the brightness of nucleus enhanced, compared with the control, indicating that Bufalin can induce the GBC-SD cell apoptosis (Fig. 2A). Western blot was performed to detect the changes in apoptosis-related proteins and the results showed that Mcl-1 was significantly down-regulated, while Bcl-2 and Bax were not significantly changed (Fig. 2B). Mcl-1 is an important anti-apoptotic protein in the Bcl-2 protein family, which can help tumor cells "evade" the drug attack and continue to grow so that tumor cells can survive, leading to the resistance of cancer patients to the chemotherapeutics.

Besides the apoptosis, Bufalin also affects the cell-cycle regulation of GBC-SD cells. The flow cytometry results showed that, after 48 h of Bufalin treatment, the ratio of GBC-SD cells at G2/M phase increased significantly, accompanied by the decrease of cells at G0/G1 phase (Fig. 2C). To explore the possible mechanism of Bufalin influencing the cell cycle, cell cycle-related proteins were detected by western blot. As shown in Fig. 2D, the expression of P21 and P27 was significantly up-regulated. These results suggested that Bufalin inhibited the proliferation of GBC-SD cells by inducing apoptosis and cell cycle arrest.

### Bufalin hindered gallbladder cancer cell invasion and migration

**Figure 3:**
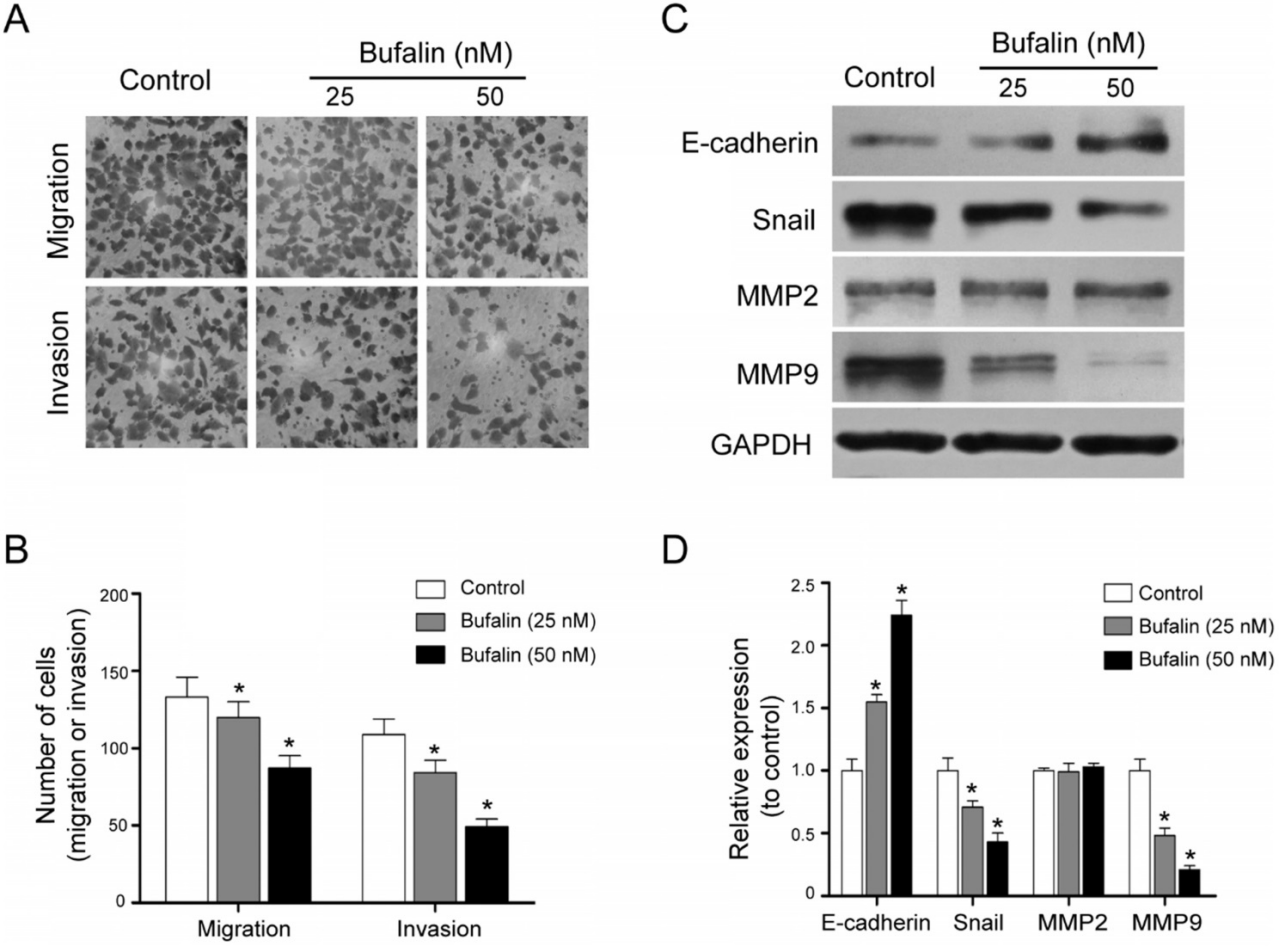
Bufalin inhibited invasion and migration of GBC-SD cells. (**A, B**) Transwell migration assay of the effects of Bufalin on migration and invasion of GBC-SD cells under different concentrations (0, 25, 50 nM, respectively) (200 ×). (**C, D**) Western blot analyzed the expression of invasion related proteins MMP2, MMP9, E-cadherin and Snail after 48 hours of treatment by Bufalin at different concentrations. GAPDH was the loading control (*P<0.05 *vs* Control).

Metastasis is the main factor for the poor prognosis of gallbladder carcinoma patients. Transwell migration and invasion assays were performed. The results indicated that Bufalin was able to hinder the cell migration and invasion (Fig. 3A and 3B). Besides, by detecting the expression of some EMT-related proteins including MMP9, MMP2, E-cadherin and Snail, we found that compared with the control, the expression of E-cadherin was up-regulated while the expression of MMP9 and Snail was down-regulated (Fig. 3C and 3D). These results suggested that Bufalin inhibited the metastasis of GBC-SD cells by suppressing the epithelial-mesenchymal transition (EMT) process.

### Bufalin repressed gallbladder CSCs development

**Figure 4:**
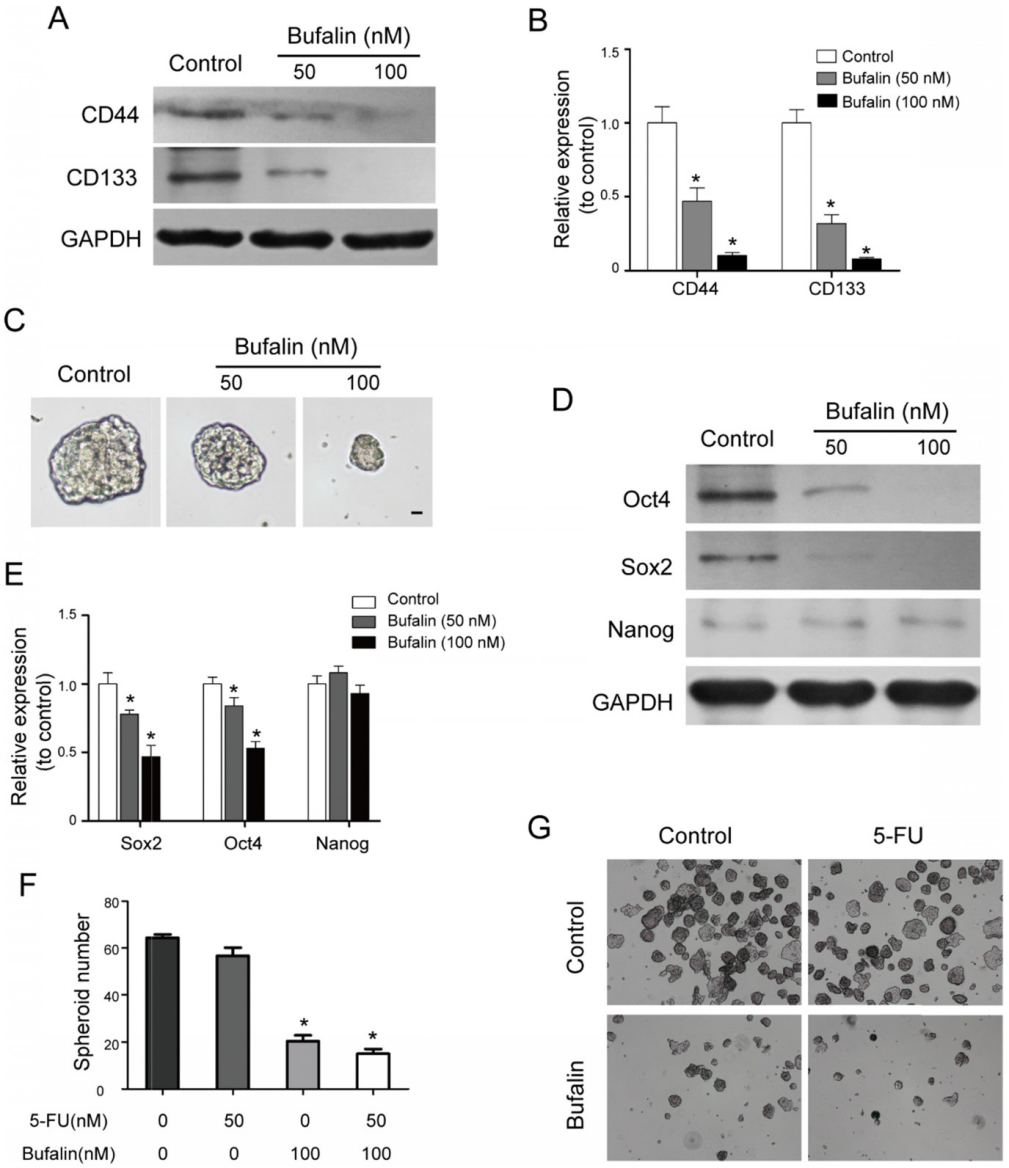
Bufalin hindered the development of gallbladder cancer stem cells. (**A, B**) Western blot analyzed the expression of stemness-associated surface proteins, CD133 and CD44 after 48 hours of treatment by Bufalin at different concentrations (0, 50, 100 nM, respectively). (**C**) Low attachment sphere-forming assay detected the cell sphere size after 48 hours of treatment by Bufalin. (**D, E**) Western blot analyzed the expression of stemness-associated proteins, Sox2, Oct4 and Nanog after 48 hours of treatment by Bufalin at different concentrations (0, 50, 100 nM, respectively). (**F, G**) Low attachment sphere-forming assay detected the cell sphere number of Bufalin on 5-FU resistant GBC-SD cells. (*P<0.05 *vs* Control).

Numerous studies have shown that tumors develop from a group of cells that have power of self-renewal and are resistant to general chemotherapy (cancer stem cells are also known as tumor initiating cells). In this study, we found that Bufalin decreased the expressing levels of stemness- associated surface proteins including CD133 and CD44 (CD133 and CD44 are considered to be the surface markers of gallbladder stem cells) by western blot (Fig. 4A and 4B). Meanwhile, the low attachment sphere-forming assay showed that Bufalin could reduce the sphere-forming size of GBC-SD cells (Fig. 4C). The self-renewal ability is one of the main features of the cancer stem cell. The western blot assay indicated that the stemness-associated factors, Sox2 and Oct4, were remarkably down-regulated, while the expression of Nanog was not significantly altered (Fig. 4D and 4E). We then detected the effect of Bufalin on GBC-SD drug-resistant cells, and found that the 5-FU resistant cells were sensitive to Bufalin and their Spheroid numbers was significantly reduced (Fig. 4F and 4G). All the results suggested that Bufalin had a certain inhibitory effect on gallbladder carcinoma stem cells.

### Bufalin inhibited the MEK/ERK and PI3-K/Akt pathways in gallbladder cancer cells

**Figure 5:**
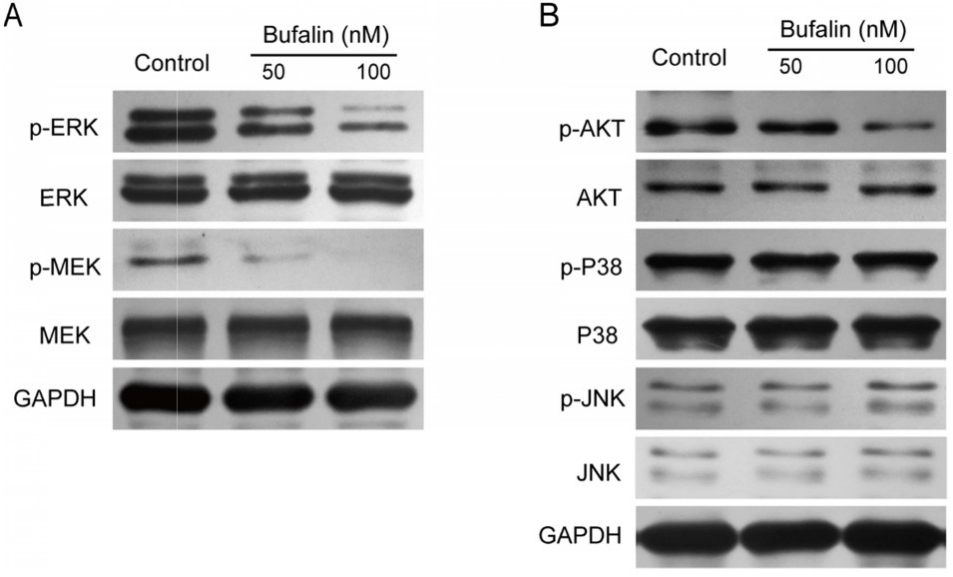
Bufalin influenced the activation of MEK/ERK and PI3K/AKT signal pathway. (**A, B**) Western blot analyzed the expression of MEK/ERK and PI3K/AKT in GBC-SD cells after 48 hours of treatment by Bufalin at different concentrations (0, 50, 100 nM, respectively).

The MEK/ERK signaling pathway plays an important role in tumor cell proliferation and metastasis. This study revealed that after Bufalin treatment, the expression of p-MEK and p-ERK in GBC-SD cells was significantly inhibited (Fig. 5A), while the expression of p-JNK and p-P38 was unchanged (Fig. 5B). It has been reported that PI3-K/Akt was closely related to the self-renewal and drug resistance of cancer stem cells 16-19. We detected the expression level of p-AKT and found that the level of p-AKT was significantly lower than that of the control (Fig. 5B), indicating that Bufalin affected the development of gallbladder cancer by influencing the PI3-K/Akt and MEK/ERK signaling pathways.

### Bufalin inhibited MEK/ERK and PI3-K/AKT signaling pathways by inhibiting the activation of c-Met

**Figure 6:**
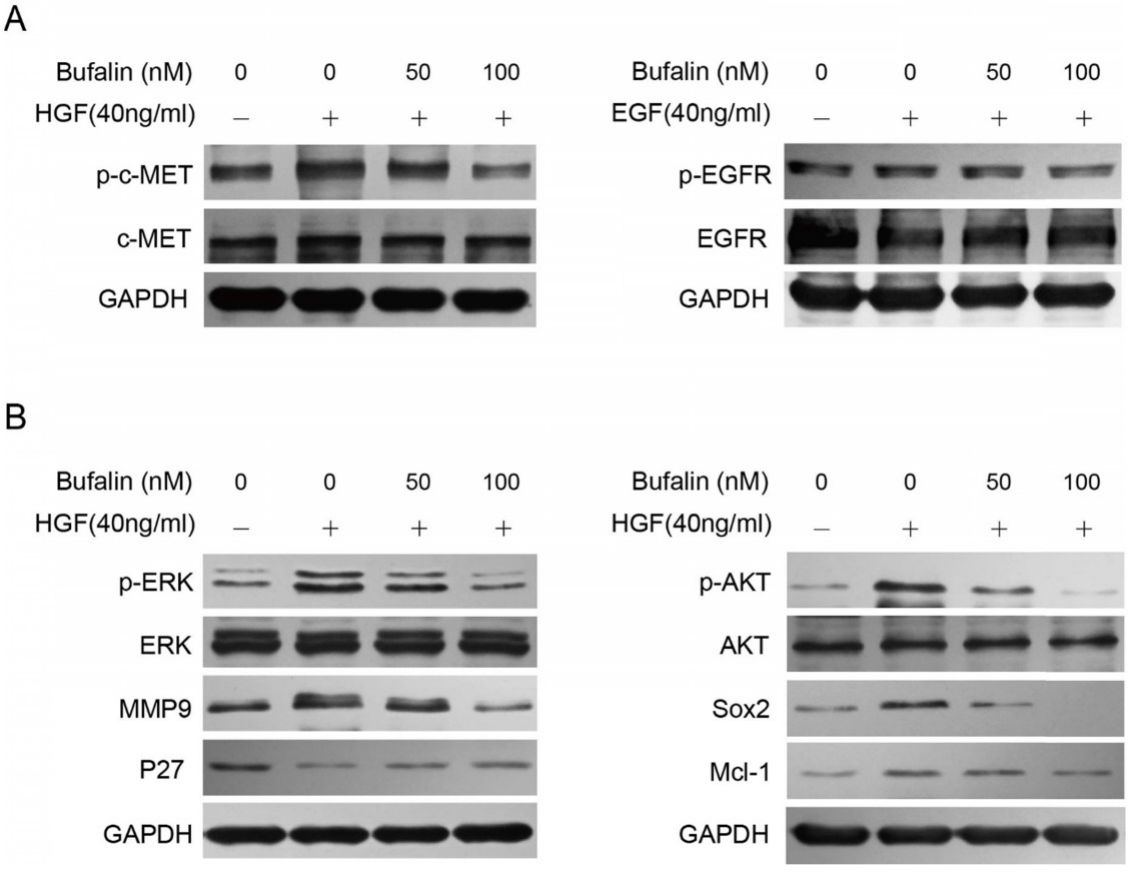
Bufalin inhibited the activation of c-MET to prohibit the development of gallbladder cancer. (**A**) Western blot analyzed the expression of p-c-MET and p-EGFR in GBC-SD cells after 48 hours of treatment by Bufalin and HGF or Bufalin and EGF combinations. (**B**) Western blot analyzed the expression of proteins associated with invasion, cell cycle and stemness in GBC-SD cells after 48 hours of treatment by Bufalin and HGF. GAPDH was used as the loading control.

The MEK/ERK and PI3-K/AKT signaling pathways are mainly regulated by RTKs (receptor tyrosine kinases), especially the EGFR and c-MET. We combined EGF and HGF respectively with Bufalin to treat the GBC-SD cells, and found that Bufalin could reduce the increasement of p-c-MET caused by HGF in a dose-dependent manner. However, Bufalin had no obvious antagonistic effect on the increase of p-EGFR caused by EGF (Fig. 6A).

Then, we treated GBC-SD cells with HGF and Bufalin for 48 h, and tested the expression of MMP9, p-ERK and P27 by western blot. According to the results, we found that expression of P27 was down-regulated while the expression of MMP9 was significantly up-regulated, compared with the cells that were treated with Bufalin specially. Similarly, the stemness-associated proteins were detected and the expressions of p-Akt, Mcl-1 and Sox2 were found to be significantly up-regulated (Fig. 6B). All the above results indicated that Bufalin decreased the activity of p-c-MET, which consequently inhibited the activation of MEK/ERK and PI3-K/AKT signaling pathways, and ultimately hindered the development of gallbladder cancer.

A drug may affect several signaling pathways in cells. We are concerned about the effect of Bufalin on gallbladder cancer stem cells. Many experiments have been carried out to identify cell phenotypes, molecular phenotypes of cancer stem cells, activation status of key molecules in the pathways, and inhibition of drugs on the activated pathways. We have explored and validated the anti-tumor activity of Bufalin by inhibiting c-MET mediated MEK/ERK and PI3K/AKT signaling pathways in gallbladder cancer.

## Discussion

Bufalin is a kind of cardiac glycosides which is derived from the serous fluid secreted by the parotid venom glands and skin of *Bufo melanostictus* and *Bufo bufo gargarizans* 20. Researches have showed that Bufalin has many pharmacological activities, including anti-inflammatory, anti-tumor, antiviral, cardiotonic and narcotic effects 21-24. Among these effects, the anti-tumor activity of Bufalin has caught the attention of many researchers. A large number of studies have indicated that Bufalin has the inhibitory effect on many kinds of malignant tumors, such as liver cancer 25, colorectal cancer 26, bladder cancer 27, gallbladder cancer 28, pancreatic cancer 29 and gastric cancer 30.

The anti-tumor mechanism of Bufalin can be summarized as follows: inhibiting the growth of tumor and angiogenesis; inducing apoptosis and differentiation; inhibiting the migration and invasion of tumor cells. Recent studies have also put forward that Bufalin has inhibitory effect on cancer stem cells (CSCs). In many cancer samples, there are a few cell subsets which have stemness and they are called as cancer stem cells (CSCs) 31, 32. Cancer stem cells were first identified in acute myeloid leukemia 33 and were gradually discovered in solid tumors including gallbladder cancer, colorectal cancer, prostate cancer, breast cancer, pancreatic cancer, liver cancer, colorectal cancer and etc. In recent years, cancer stem cells have been an intense cancer research focus. Many studies have shown that CSCs play a crucial role in tumor formation, recurrence, metastasis and chemotherapy resistance.

Self-renewal capability is the most basic characteristic of CSCs. Shi et al. developed CD133^+^ cell spheres from primary human gallbladder cancer cells and GBC-SD cells using the serum-free suspension culture method 2. These cell spheres have a strong capacity of proliferation *in vitro* and can form subcutaneous xenograft in nude mice, revealing their self-renewal characteristics as the cancer stem cell.

CSCs can promote cancer cell metastasis. Cancer metastasis is a crucial factor that leads to poor therapeutic effect and prognosis. It involves a complex series of steps and the epithelial-mesenchymal transition (EMT) is one of the most important stage in metastasis. EMT is a biological process that the differentiated epithelial cells obtain a migratory characteristic like the cells while lose the typical epithelial characteristics. Moon, et al. found that overexpressing CD133 could up-regulate the expression of N-cadherin and vimentin, while down-regulate the expression of E-cadherin to promote the development of EMT and enhance the invasion and migration abilities of squamous cell carcinoma 34. Li et al. reported that the mesenchymal cell marker, vimentin, was expressed in the progenitor cell of highly metastatic colon cancer but not in the progenitor cell of non-metastatic pancreatic cancer, indicating that EMT is correlated with the CSCs metastasis 35.

CSCs promote drug resistance of cancer cells to chemotherapy. Traditional tumor chemotherapy is targeted at proliferating cells. The faster tumor cells proliferate, the more sensitive they are to drugs. Ricci-Vitiani et al. found that about 96% of the leukemia patients had CSCs at G0 phase, and the traditional chemotherapy was inefficient in these cases 36. Traditional radiotherapy and chemotherapy are effective to differentiated, mature cancer cells, leukemia CSCs at G0 phase do not proliferate, so they are hard to be fully eliminated by chemotherapy. Shi et al. also found that, the CD44^+^ and CD133^+^ gallbladder CSCs have tolerance ability of gemcitabine and 5-fluorouracil 37. The mechanism in CSCs may be that, the intracellular drugs were actively pumped out using the energy from ATP by influencing the expression of multidrug resistance-associated protein ABCG2 and transcription factor Gli1, so as to reduce the intracellular drug level and to protect themselves from the damage of cytotoxic drugs. That's why most cancer patients are still prone to metastasis and recurrence after chemotherapy, which is a big threat to their survival.

The role and molecular mechanism of Bufalin in inhibiting cancer stem cells have been reported in only a few cases of osteosarcoma and pancreatic cancer. In this study, we showed that Bufalin had a certain inhibitory effect on the growth, invasion and migration of GBC-SD cells. Moreover, we found that Bufalin had an inhibitory effect on the sphere-forming ability of gallbladder cancer. Western blot results suggested that Bufalin decreased the expression of stemness-associated surface proteins CD133 and CD44, and stemness-associated factors Sox2 and Oct4, which further illustrated that Bufalin has the inhibitory effect on gallbladder cancer stem cells. Mechanism research found that Bufalin inhibited the activation of MEK/ERK and PI3-K/AKT signaling pathways by inhibiting the expression of p-c-Met, thus inhibiting proliferation of GBC-SD cells and reducing the self-renewal ability of gallbladder cancer stem cells, which further increased the radiation and chemotherapy sensitivity, and reduced the recurrence and metastasis of gallbladder cancer.
